# A congenital case of vascular haemangioma of the right hand

**DOI:** 10.11604/pamj.2024.49.136.46020

**Published:** 2024-12-31

**Authors:** Shiwani Padmakar Dandade, Arati Raut

**Affiliations:** 1 Medical-Surgical Nursing Department, Smt. Radhikabai Meghe Memorial College of Nursing, Sawangi (Meghe), Wardha, Maharashtra, India

**Keywords:** Haemangioma, congenital, arteriovenous aneurysm, malformations

## Image in medicine

A 10-month-old girl's mother complained of the baby having multiple swells on her right hand ever since she was born. According to the mother's account, they observed that the right hand had numerous swellings as soon as she was born. The swelling has been growing since then. The swelling grew larger, and more lesions appeared over time. Itching or pain is not linked to swelling. All vaccinations are administered to the expectant mother during her pregnancy, except the nine-month vaccination. According to the birth history, a lower segment caesarean section was performed on a 3.5 kg female child. After a few months of treatment, she was diagnosed with a congenital vascular haemangioma of the right hand. Vascular abnormalities are the most frequent congenital and neonatal abnormalities that children encounter. Most often, benign vascular lesions are haemangiomas. The majority of them are incidentally found and exhibit no symptoms. Tumours and deformities of all kinds are considered vascular anomalies. For managing the right hand, vascular haemangioma is treated with a sclerotherapy procedure. Sclerotherapy is a procedure used to treat lymphatic system and blood vessel malformations. The vessels are injected with a medication, which causes them to constrict. It is used for children and young adults with vascular or lymphatic malformations. In these cases, 50 ml of sclerotherapy injection is injected.

**Figure 1:**
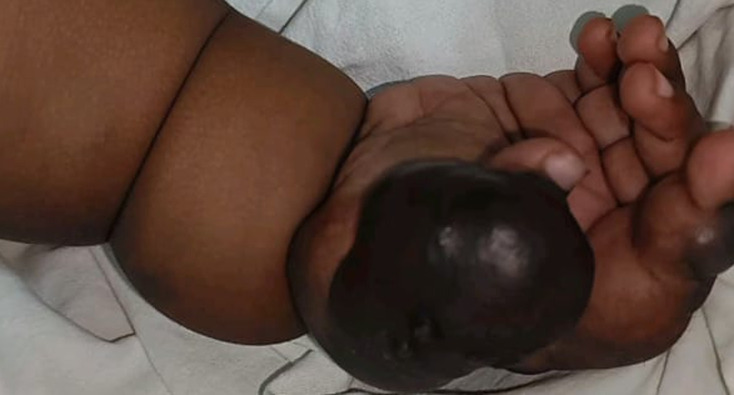
vascular haemangioma of the right hand

